# Placental Delta-Like 1 Gene DNA Methylation Levels Are Related to Mothers' Blood Glucose Concentration

**DOI:** 10.1155/2019/9521510

**Published:** 2019-12-11

**Authors:** Bai-Hui Zhao, Ying Jiang, Hong Zhu, Fang-Fang Xi, Yuan Chen, Ye-Tao Xu, Fang Liu, Ya-Yun Wang, Wen-Sheng Hu, Wei-Guo Lv, Qiong Luo

**Affiliations:** ^1^Department of Obstetrics, Women's Hospital, Zhejiang University School of Medicine, Hangzhou, China; ^2^Department of Obstetrics and Gynecology, The International Peace Maternity and Child Health Hospital of China Welfare institute, Shanghai, China; ^3^Department of Obstetrics and Gynecology, The First Affiliated Hospital of Nanjing Medical University, Nanjing, China; ^4^Department of Cardiology, Beijing Anzhen Hospital, Capital Medical University, Beijing, China; ^5^Department of Obstetrics, Maternal and Child Health Care Hospital, Hangzhou, China

## Abstract

**Purpose:**

We aim to identify the methylation status of delta-like 1 (DLK1) in the placenta and the correlation between DLK1 methylation and maternal serum glucose level and fetal birth weight.

**Methods:**

We analyzed the gene expression of DLK1 gene in both maternal and fetal sides of the placenta in a GDM group (*n* = 15) and a control group (*n* = 15) using real-time polymerase chain reaction. With MethylTargetTM technique, we detected the methylation status of DLK1 promotor in the placenta. Furthermore, Pearson's correlation was used to confirm the association of methylation alteration of DLK1 promoter and maternal 2 h OGTT glucose level and fetal birth weight.

**Results:**

In our study, we found that DLK1 expression in both maternal and fetal sides of the placenta decreased significantly in GDM group compared with control group, and it was caused by hypermethylation of DLK1 promoter region. Additionally, the methylation status of DLK1 gene in the maternal side of the placenta highly correlated with maternal 2 h OGTT glucose level (coefficient = 0.7968, *P* < 0.0001), while the methylation status in the fetal side of the placenta was closely related to fetal birth weight (coefficient = 0.6233, *P* < 0.0001).

**Conclusions:**

Our results demonstrated that altered expression of DLK1 was caused by the hypermethylation of DLK1 promoter region in the placenta, and intrauterine exposure to GDM has long-lasting effects on the epigenome of the offspring.

## 1. Introduction

Gestational diabetes mellitus (GDM) is defined as the development of high blood glucose levels during pregnancy in women without diabetes [1]. The population of GDM has increased over time worldwide [2]. In line with the fetal programming hypothesis, offspring exposed to women with GDM have a higher rates of type 2 diabetes [3], obesity [4], and cardiovascular diseases [5] in their childhood and adulthood. Of interest, these effects have transgenerational transition, suggesting that exposure to an adverse fetal environment may affect multiple generations [6, 7]. Consequently, intrauterine hyperglycemia becomes a major public health problem.

Multiple hypotheses have been proposed to explain how intrauterine hyperglycemia would aroused those effects on GDM offspring, such as perpetual malorganization of the hypothalamic regulation centers induced metabolism disruption [8]; maternal hyperglycemia leads to increasing fetal insulin secretion which is closely related to macrosomia and later obesity [9], even though the molecular mechanism is still unclear. Nevertheless, epigenetic modifications open a new path to uncover these associations, in particular DNA methylation [10]. The most widely defined DNA methylation is the covalent addition of the methyl group at the 5-carbon of the cytosine ring making for 5-methylcytosine (5-mC), affecting gene expression [11]. Lower methylation levels of adiponectin gene (ADIPOQ) on the fetal side of the placenta were related to higher maternal glucose levels [12]. Whole-Genome Methylation of GDM placenta showed 3271 genes differentially methylated compared with that of normal placenta; these genes were likely involved in the metabolic disease pathway and associated with newborn weight, such as VIPR1 (vasoactive intestinal peptides receptors) in the placenta which might be related to maternal glucose metabolism and TRIB1 (Tribbles-1) which correlates with lipid/energy homeostasis [13].

Placenta is a unique tissue which plays an indispensable role in fetal growth and nutrient exchange and meanwhile exposed to the same intrauterine environment as the fetus. Placental growth is most vulnerable to maternal intrauterine environment during peri-implantation period and the first trimester of gestation [14]. Epigenetics, especially imprinted genes have been proposed as nutrient sensors regulating placental growth and nutrient transport to the fetus, subsequently stimulating or constraining fetal growth. Recently, reduced methylation level of the adiponectin gene in fetal placenta has been proven to link with mothers' blood glucose concentration [12]; decreased MEST methylation levels may contribute to obesity predisposition throughout life [15].

Delta-like 1 (DLK1) is a maternal imprinted gene, encoding a transmembrane protein Notch/serrate/delta family [16], suppression of Notch signaling leading to an improvement in insulin sensitivity with reduced blood glucose levels [17]. Increasing evidence obviously indicates that DLK1 is an inhibitory regulator of Notch signaling by interacting with Notch receptor Notch1 [18]. Furthermore, the ectodomain cleavage of DLK1 inhibited adipogenesis in vitro and in vivo [19, 20].

Although previous studies have indicated a relationship between DLK1 and diabetes and obesity, limited data specifying the link between the methylation status of DLK1 promoter and GDM development has been published. Thus, in our study, we tested whether the methylation status of DLK1 promotor in placental tissues is associated with maternal glucose levels during pregnancy.

## 2. Material and Methods

### 2.1. Subjects

GDM is diagnosed if at least one value of plasma glucose concentration is equal to or over the thresholds of 92 mg/dl, 180 mg/dl, and 153 mg/dl (fasting and one-hour and two-hour postglucose load values comparatively), after 75 g oral glucose tolerance test (OGTT) between gestational weeks 24 and 27 [21, 22]. After the diagnosis, women obtained dietary counseling by an obstetrician. BMI was measured according to the standardized procedures of the Airlie conference in the first and third trimesters. Children's birth weights were detected at the timing of delivery; APGAR scores of all the children are 10-10 (1 min-5 min).

Fifteen normal pregnant women and compared fifteen GDM pregnant women are included in our study. We excluded children born with cardiovascular diseases, premature delivery, and congenital anomalies and mothers with other disorders, such as pre-GDM, polycystic ovarian syndrome, uncontrolled thyroid disorders which were known to affect glucose metabolism, and other gestational complications such as preeclampsia and intrahepatic cholestasis.

The Ethics Committee of Women's Hospital, School of Medicine, Zhejiang University (Hangzhou, China), approved this study, and written informed consent was obtained from one of the parents of the children and from the women whose placenta was collected. The investigation conforms to the principles outlined in the Declaration of Helsinki.

### 2.2. Placental Tissue Sampling

We collect the placenta from both fetal and maternal sides in less than 15 minutes after delivery by well-trained clinicians, and this was kept at -80°C until nucleic acid and genomic DNA extraction [12]. Analyses were performed on both sides separately.

### 2.3. RNA Isolation and Gene Expression (Quantitative PCR)

Placental RNA was extracted using TRIzol Reagent (Invitrogen Life Technologies, Carlsbad, CA, USA) according to the manufacturer's protocol. DNA contamination was wiped out by DNase I digestion (AM2222, Thermo Fisher Scientific). Total RNA was reverse-transcribed from 1 *μ*g of RNA using oligo dT and random primers (TaKaRa, Japan) and amplified by SYBR® Premix Ex Taq™ (TaKaRa, Japan) in the ABI Prism 7900HT (Applied Biosystems, Foster City, CA). All threshold cycle (Ct) values of each sample were utilized in the post-PCR data analysis. GAPDH is our internal control, and gene expression levels were normalized against GAPDH. Full list of primer sequences is shown in Supplemental [Supplementary-material supplementary-material-1].

### 2.4. DNA Isolation, Bisulfite Conversion, and Methylation Analysis by MethylTargetTM

Total genomic DNA was isolated from placenta tissues by using the Genomic DNA Purification Kit (Invitrogen, cat. K05112, USA). Bisulfite was converted using the EpiTect Bisulfite Kit (QIAGEN, Valencia, CA) according to the manufacturer's instructions to deaminate cytosine to uracil; 5-methyl-cytosine was protected from deamination. We selected two CpG sites in the proximal promoter of DLK1 gene (Supplementary [Supplementary-material supplementary-material-1]). Then, 2 *μ*l bisulfite DNA was prepared for multiplex PCR reaction in 20 *μ*l reaction mixture, including 1x reaction buffer (Takara), 1 U HotStarTaq polymerase (Takara), 3 mM Mg2+, 0.2 mM dNTP, and 0.1 *μ*m of each primer for PCR amplification. The cycling program was as follows: 95°C for 2 min; 11 cycles of 94°C for 20 s, 63°C decreasing 0.5°C per cycle for 40s, and 72°C for 1 min; subsequently 24 cycles of 94°C for 20 s, 65°C for 30 s, and 72°C for 1 min; and 72°C for 2 min. Then, 1 *μ*l diluted PCR amplicons were used for index PCR reaction in 20 *μ*l mixture, containing 1x reaction buffer (NEBQ5™), 1 U Q5™ DNA polymerase (NEB), 0.3 mM dNTP, 0.3 mM of F primer, and 0.3 *μ*M of index primer, which amplified in cycling program as follows: 98°C for 30 s; 11 cycles of 98°C for 10 s, 65°C for 30 s, and 72°C for 30 s; and 72°C for 5 min. QIAquick Gel Extraction Kit (QIAGEN) was utilized to purify PCR amplicons (170-270 bp) and then loaded onto Illumina NextSeq 500 (Illumina, San Diego, CA, USA) according to the manufacturer's protocols.

### 2.5. Statistical Analysis

Quality control of sequencing reads was performed by FastQC. Filtered reads were mapped to genome by BLAST after read recalibration with USEARCH. Data were analyzed using SPSS 25.0 and were presented as mean ± SE or mean ± SD. Statistical analysis includes unpaired two-tailed Student's *t*-test, as described in the figure legends or Excel legends. Correlations of the methylation profiles with maternal 2 h OGTT glucose levels and birth weight were performed using the Pearson correlation coefficient; *P* < 0.05, *P* < 0.01, or *P* < 0.001 was considered statistically significant.

## 3. Results

### 3.1. Baseline Characteristics

Maternal and offspring characteristics including OGTT results are presented in Supplementary [Supplementary-material supplementary-material-1]. On average, there was no significance in the aspect of maternal age, BMI, and weight gain. OGTT results showed that GDM patients had higher concentrations of glucose at fasting and 2 h point (5.34 ± 1.45 vs. 4.13 ± 0.51, *P* < 0.01; 8.56 ± 1.78 vs. 5.76 ± 1.49, *P* < 0.01). Furthermore, birth weight of offspring of GDM group is much larger than control group (3.61 ± 0.25 vs. 3.20 ± 0.43, *P* < 0.01), which is in line with previous studies [23].

### 3.2. Decreased Expression of DLK1 in GDM Placenta Both Maternal and Fetal Sides

**Figure 1:**
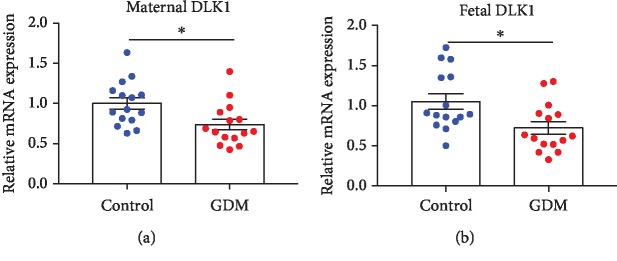
The mRNA expression of DLK1 in both maternal and fetal sides of the placenta. (a) DLK1 expression in the maternal side of the placenta. (b) DLK1 expression in the fetal side of the placenta. RNA levels determined by RT-PCR. Data were analyzed with the Eq. 2^-*△△*CT^, where △△CT = △CT (treatment group)‐△CT (control group) and △CT = △CT (sample)‐△CT (internal control). The values were normalized to GAPDH mRNA levels. Control group has 15 samples, and GDM group has 15 samples. In all panels, data are presented as mean ± SE, ^∗^*P* < 0.05, and ^∗∗^*P* < 0.01. Significance was determined by Student's *t*-test.

In order to confirm whether intrauterine hyperglycemia (GDM group) alters DLK1 mRNA level in the GDM placenta, real-time quantitative RT-PCR was performed. We found that in both maternal and fetal sides of the placenta, DLK1 expression significantly decreased in GDM groups compared with control groups (Figures 1(a) and 1(b)).

### 3.3. Increased DNA Methylation of DLK1 Promoter in GDM Placenta Both Maternal and Fetal Sides

**Figure 2:**
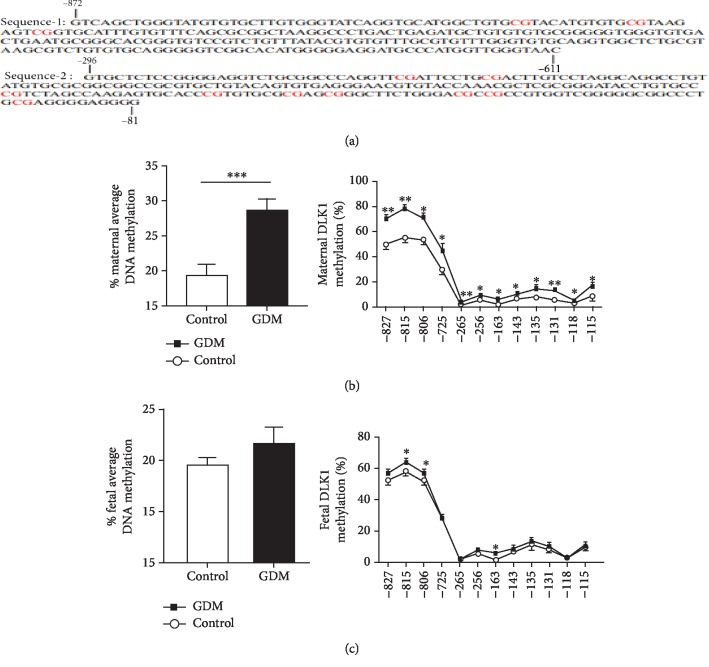
Methylation analysis of DLK1 promotor region by MethylTargetTM. (a) Detailed sequences of two regions in DLK1 promotors which included 38 CpGs. (b, c) Methylation status of DLK1 promotor region in maternal and fetal sides of the placenta, including average DLK1 methylation level, and special individual CpG sites. In all panels, data are presented as mean ± SE, ^∗^*P* < 0.05, ^∗∗^*P* < 0.01, and ^∗∗∗^*P* < 0.001. Significance was determined by Student's *t*-test.

A total of 38 CpGs located in the DLK1 promoter regions were epigenotyped. We illustrated CpG islands and the results in Figure 2. We found that nine CpGs in the maternal sides of the GDM placenta showed a higher methylation compared with control placenta, while in the fetal side of the GDM placenta (Figure 2(b)), only three CpGs increased methylation levels (Figure 2(c)). From the aspect of the mean DNA methylation levels, maternal placenta sides indicated an obviously higher methylation (28.75% ± 1.50% vs. 19.30% ± 1.67%, *P* = 0.0005) (Figure 2(b)); although there was an increased methylation tendency in fetal sides of the placenta of the GDM group, no significance was found (21.61% ± 1.71% vs. 19.40% ± 0.90%, *P* = 0.2806) (Figure 2(c)).

### 3.4. Correlation between Maternal Glucose Levels, Birth Weight, and DNA Methylation Levels in Placental Tissues

**Figure 3:**
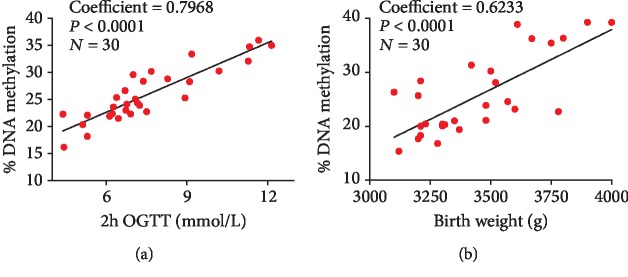
Spearman correlation between placental DLK1 gene promotor DNA methylation and 2 h OGTT glucose levels and fetal birth weight. (a) The correlation between the methylation level of DLK1 promotor region in the maternal side of the placenta and 2 h OGTT glucose levels. (b) The correlation between the methylation level of DLK1 promotor region in the fetal side of the placenta and fetal birth weight. 30 samples were included in the analysis (15 control samples and 15 GDM samples).

A higher level of maternal glucose at 2 h OGTT was significantly associated with higher mean DNA methylation at DLK1 promotor region on the maternal side of the placenta (coefficient = 0.7968, *P* < 0.0001) (Figure 3(a)). Besides, increased mean DNA methylation of DLK1 promotor region in the fetal side of the placenta was moderately connected with higher birth weight (coefficient = 0.6233, *P* < 0.0001) (Figure 3(b)).

## 4. Discussion

Barker's hypothesis considered that increased risk of obesity, cardiovascular diseases, and diabetes in childhood and adulthood originated from fetal exposure to adverse environments such as intrauterine nutritional deficiency and impaired glucose tolerance [24]. Nevertheless, the molecular mechanisms linking fetal life and the long-term adult chronic diseases are still poorly understood. Our study focused on the effect of intrauterine hyperglycemia on the newborn epigenetic profile. The most crucial finding was that placental DNA methylation is associated with mother's glucose levels during pregnancy and newborns' birth weight. Interestingly, the DLK1 DNA methylation in the maternal side of the placenta indicated the strong correlation with 2 h glucose levels, and the DLK1 DNA methylation in the fetal side of the placenta significantly connected with birth weight, which has already been reported that large birth weight (LBW) is a risk factor of obesity on childhood [25].

DLK1 is a paternal-expressed, maternal-imprinted gene, which has two forms of protein, soluble form of DLK1 cleaved by a tumor necrosis factor alpha converting enzyme (TACE) [19] and protein remains on the cell membrane. It is considered as a growth factor, maintaining the proliferative state of undifferentiated cells, thus highly expressed on embryonic tissues and lower expressed on adult tissues [26, 27]. Furthermore, transgenic mice with overexpression of DLK1 in adipose tissue showed a substantial decreased in total fat weight [28] and decreased insulin sensitivity [29]. Increased expression of DLK1 could act primarily on the growth hormone (GH) axis to shift the metabolic mode to fat acid oxidation, which has beneficial effects on hepatic lipid deposition and protects from steatosis [30]. Soluble DLK1 protein is significantly lower in fetuses of GDM group compared to normal pregnancies [31], and in our study, we have found that placental DLK1 expression also decreased in GDM group, which indicated that two forms of protein exhibited the same tendency in intrauterine hyperglycemia environment. In vivo, we had previously found that placental DLK1 expression decreased in GDM mice compared with normal pregnancy, which had intergenerational effects [32]. Due to increased insulin-like growth factor I (IGF I) competitively bound to IGF I R [33], DLK1 failed to activate ERK1/2 signaling [18], which might be one potential mechanism for excess adiposity in GDM offspring [34].

It has been reported that the DNA methylation plays a critical role in fetal and placenta development [35, 36]. At first, we showed that the DNA methylation within DLK1 gene promoter was significantly hypermethylated in both maternal and fetal sides of the placenta of mothers with GDM in comparison to normal glucose tolerance mothers. Of interest, we have found that 2 h OGTT glucose level is positively correlated with the maternal DLK1 DNA methylation profile, which indicated the importance of detecting glycemic levels in response to glucose intake for diagnosis and treatment of GDM. Previous studies also reported that lower mean methylation of adiponectin gene [12] and lipoprotein lipase (LPL) gene [37] were correlated to 2 h OGTT glucose level. GDM is a complicated metabolic status during pregnancy, it is clear that fetal development and growth are affected by the full extent of glucose levels throughout pregnancy period, without a certain threshold value. Furthermore, higher maternal glucose concentrations linked to excess adiposity in childhood and adulthood [34], suggesting that there was an adverse long-term effect on offspring whose mother with a slightly high serum glucose concentration. Besides, in our study, the hypermethylation status of DLK1 promotor region in the fetal side of the placenta was significantly associated with large birth weight (LBW). LBW, particular very high, increases the risk of type 2 diabetes and obesity in adulthood [38, 39]. Therefore, a more inclusive OGTT cutoff value should be evaluated and extended, which might reduce as much as possible hyperglycemia deleterious effects on offspring's health.

The population of women influenced by hyperglycemic disorders in pregnancy increases every year all around the world, which accompanied by increased risk of obesity and insulin resistance in offspring, which forms a vicious circle. Our results showed that the DLK1 methylation was associated with 2 h OGTT glucose levels and birth weight, suggested that the epigenetic alteration around DLK1 could be one potential mechanism involved in fetal programming of obesity and other metabolic disorders in childhood and adulthood. Our study provides a new gene target on the epigenetic regulation, and studies about other genes concerning to glycemic regulation, insulin resistance pathway, and lipid metabolism will be essential to confirm our findings.

Although our finding was generated in limited GDM patients, it is important to recognize that epigenetic alteration of imprinting gene DLK1 could be one potential mechanism involved in fetal programming of growth. These altered DNA methylation markers not only partly explained the obesity predisposition in GDM offspring but more importantly will hopefully allow for the early diagnosis and therapy of individuals with a propensity for adult-onset disease. Epigenetic modification may participate in genesis and development of diverse diseases. The main limitation of our study is the small number of sample size; the large-scale validation is necessary to confirm and generalize our preliminary results.

In conclusion, we provide the first evidence that DNA methylation change of the DLK1 gene on the maternal side of the placenta is associated with maternal 2 h OGTT glucose levels, while the DLK1 methylation status on the fetal side of the placenta is correlated with fetal birth weight. This might afford a potential mechanism contributing to obesity predisposition throughout life.

## Data Availability

All data generated or analyzed during this study are included in this published article.

## Supplementary Materials

Supplementary Materials We have listed three tables in the supplementary material files. Supplementary Table 1: the characteristic of children at birth and the maternal characteristics. Supplementary Table 2: nucleotide sequences of primers (human) used for real-time quantitative PCR (SYBR Green). Supplementary Table 3: nucleotide sequences of primers (human) used for MethylTarget sequencing.
