# Supplementary material for: Placental Delta-Like 1 Gene DNA Methylation Levels Are Related to Mothers' Blood Glucose Concentration
Source: J Diabetes Res. 2019 Dec 11;2019:9521510. doi: 10.1155/2019/9521510 (PMC6927055; doi:10.1155/2019/9521510)
Supplement: Supplementary Materials — We have listed three tables in the supplementary material files. Supplementary Table 1: the characteristic of children at birth and the maternal characteristics. Supplementary Table 2: nucleotide sequences of primers (human) used for real-time quantitative PCR (SYBR Green). Supplementary Table 3: nucleotide sequences of primers (human) used for MethylTarget sequencing. [file 9521510.f1.pdf]

Supplementary Table 1. The characteristic of children at birth and the maternal characteristics.

**Characteristics**	**Control(n=15)**	**GDM(n=15)**
Maternal age (years)	31.80 ± 3.60	32.80 ± 4.36
First trimester BMI (kg/m^2)	22.26 ± 2.30	22.70 ± 2.23
Third trimester BMI (kg/m^2)	26.59 ± 3.23	27.34 ± 2.86
Weight gain (%)†	26.16 ± 5.28	25.41 ± 8.98
Fasting glucose levels (mM)	4.13 ± 0.51	5.34± 1.45**
2h-OGTT glucose levels (mM)	5.76 ± 1.49	8.56 ± 1.78**
Birth weight (kg)	3.20 ± 0.43	3.61 ± 0.25**

Data are presented as Mean ± SD.

* *P* <0.05; ** *P* <0.01 vs. Control.

† weight gain between the first trimester and third trimester (% of initial body weight)

Supplementary Table 2. Nucleotide sequences of primers (human) used for real-time quantitative PCR (SYBR Green)

Target RNA	Primers (5' to 3' direction)	Product Size	GeneBank Acc.
GAPDH	GGGAAACTGTGGCGTGAT	308bp	NM_008084
	AAGGTGGAGGAGTGGGTGT		
Dlk1	CTGAAGGTGTCCATGAAAGAG	273bp	NM_001317172.1
	GCTGAAGGTGGTCATGTCGAT		

Supplementary Table3 Nucleotide sequences of primers (human) used for MethylTarget Sequencing

Methylation Analysis	Forward Primer	Reverse Primer	Product
Dlk1-1st Primer	GTTAGTTGGGTATGTG TGTTTGTG	ATTACCCAACCATAAAC ATCCTC	262bp
Dlk1-2nd Primer	GTGTTTTTYGGGGAGG TTTG	CCCCTCCCCTCRCAAAA C	216bp
